# Comment on “ApoE e4e4 Genotype and Mortality With COVID-19 in UK Biobank” by Kuo et al

**DOI:** 10.1093/gerona/glaa202

**Published:** 2020-08-17

**Authors:** Dimitri A Nikogosov, Artem D Shevlyakov, Ancha V Baranova

**Affiliations:** 1 RnD Department, Atlas Biomed Group Limited, London, UK; 2 Research Center for Medical Genetics, Moscow, Russia; 3 School of Systems Biology, George Mason University, Fairfax, Virginia

We would like to comment on the two studies performed by Kuo et al (1,2). We have read with great interest both papers which described an association between the epsilon alleles of the *APOE* gene and the severity and the mortality rate of COVID-19. Initially, the authors showed that APOE*E4/*E4 homozygotes were more likely to be SARS-CoV-2 positive when compared to APOE*E3/*E3 homozygotes, regardless of presence of APOE*E4-associated diseases (dementia, hypertension, coronary artery disease, type 2 diabetes) (1). In the second study of COVID-19 mortality, authors reported an association between APOE*E4/*E4 homozygous status and increased risks of mortality with test-confirmed COVID-19 when compared to APOE*E3/*E3 homozygotes.

We fully agree that preexisting comorbidities greatly influence COVID-19 severity and mortality (3) and raise interest in the association between the underlying genetic component of comorbidities and COVID-19. On the one hand, this approach conveniently narrows the search field and allows a deep dive into the pathophysiology and the genetics at the intersection of particular comorbidity and COVID-19. On the other hand, such “hypothesis-aware” approaches introduce a bias for specific loci, genes, or sequence variations, while excluding other, possibly more significant, associations. When applied to the field of genetic association studies, hypothesis-aware approach greatly relaxes the threshold of the significance for detected associations by removing a necessity for an adjustment for multiple testing. In contrast, “hypothesis-free” approach of the genome-wide association studies sets the threshold for significance at 5 × 10^−8^ with an allowance for minor fluctuations depending on the methodology (4–6). The exact value of a threshold is highly debatable; however, recent studies have shown that an even stricter significance criterion should be applied (6).

An important principle of evidence-based approach is to select criteria for the study success before running the analyses rather than after the fact. Unfortunately, the initial paper by Kuo et al (1) does not provide any fixed threshold for statistical significance or any indication that this threshold was selected beforehand. The authors report the obtained *p* values, ranging from 2.42 × 10^−7^ to 8.21 × 10^−5^, and state that the APOE*E4/*E4 combination increases the risk of severe COVID-19 infection. However, since the threshold was not preconditioned, the conclusion about the significance of the reported variants remains questionable. According to the current state of art in genome-wide association studies, only variants with *p* values < 5 × 10^−8^ may be considered significant. One may argue that for one-locus designs such a strict threshold is unnecessary because multiple testing does not take place; however, in our opinion, such practice sets a dangerous precedent where a prior internal genome-wide association screening may highlight a few candidate variants which are then cherry-picked and reported in a manner of non-genome-wide but plain association study. Moreover, the exact number of independent sequence variations in the human genome does not change whether a single or several million single nucleotide variations (SNVs) are tested. In this light, it looks like a genome-wide *p* value threshold of at least 5 × 10^−8^ should be mandatory for any reported genetic association regardless of the number of SNVs tested.

The subsequent paper by Kuo et al (2) resolves our threshold concerns only partially. The *p* values of the observed association between APOE*E4/*E4 homozygotes and COVID-19 positivity indeed ranged from 1.23 × 10^−9^ to 2.10 × 10^−7^. This association now seems significant despite the fact that independent replication is still required, in accordance with the well-established practice of genome-wide association studies (7). However, an association between *APOE* alleles and COVID-19 mortality was reported with *p* values in range from 3.08 × 10^−7^ to 3 × 10^−3^ (2), which is still substandard.

We believe that the design of both studies (1,2), which allowed Kuo et al to find mentioned associations, requires closer scrutiny. As of April 26, 2020, among nearly 500 000 participants of the UK Biobank, only 1474 were tested for SARS-CoV-2. Of these, 622 participants showed at least one positive test result. These people were assigned to the case group in the first study by Kuo et al (1). The control group was composed of negative or untested participants excluding those who died before the epidemic (*n* = 15 885) and consisted of 223 056 people. The second paper by Kuo et al (2) used a similar approach.

We are deeply concerned that in both of the studies a cohort of untested participants was assumed to represent a mild course of COVID-19. The authors compared the genotypes of participants who were positive for SARS-CoV-2 (and, in the second study, died) with genotypes of participants with an unknown COVID-19 status, plus a small fraction of SARS-CoV-2 negatives (0.37% of the overall control group in the first study). No tangible proof was proffered to indicate that the individuals with unknown COVID-19 status were not severely affected, or would not be severely affected in the future.

In our opinion, a different study design would be more proper. The initial release of the COVID-19 data set by UK Biobank included not only the SARS-CoV-2 test results but other pertinent information as well, including evidence whether a participant was an inpatient or not. We think that the case and control groups should consist of SARS-CoV-2 positive participants only, after splitting by the origin of their test results. The participants who tested positive during a hospital stay should be considered to have a severe form of COVID-19, and the participants who had all the positive test results obtained outside a hospital setting should be considered to have a mild form of the infection. Such design excludes any uncertainty regarding SARS-CoV-2 presence and gives a more explicit designation of the severity of disease, though the fact of hospitalization is still a proxy for the true course of COVID-19.

Further investigations are warranted for uncovering the biological mechanisms linking *APOE* haplotypes to COVID-19 severity and mortality as Kuo et al conclude, but these findings should be first confirmed in a more robust study design.
